# Complementing the Sugar Code: Role of GAGs and Sialic Acid in Complement Regulation

**DOI:** 10.3389/fimmu.2015.00025

**Published:** 2015-02-02

**Authors:** Alex Langford-Smith, Anthony J. Day, Paul N. Bishop, Simon J. Clark

**Affiliations:** ^1^Wellcome Trust Centre for Cell-Matrix Research, Faculty of Life Sciences, University of Manchester, Manchester, UK; ^2^Centre for Hearing and Vision Research, Institute of Human Development, University of Manchester, Manchester, UK; ^3^Centre for Advanced Discovery and Experimental Therapeutics, University of Manchester and Central Manchester University Hospitals NHS Foundation Trust, Manchester, UK; ^4^Manchester Academic Health Science Centre, University of Manchester and Central Manchester University Hospitals NHS Foundation Trust, Manchester, UK; ^5^Manchester Royal Eye Hospital, Central Manchester University Hospitals NHS Foundation Trust, Manchester, UK

**Keywords:** sialic acid, heparan sulfate, glycosaminoglycan, complement factor H, properdin, innate immunity, tissue specificity, complement regulation

## Abstract

Sugar molecules play a vital role on both microbial and mammalian cells, where they are involved in cellular communication, govern microbial virulence, and modulate host immunity and inflammatory responses. The complement cascade, as part of a host’s innate immune system, is a potent weapon against invading bacteria but has to be tightly regulated to prevent inappropriate attack and damage to host tissues. A number of complement regulators, such as factor H and properdin, interact with sugar molecules, such as glycosaminoglycans (GAGs) and sialic acid, on host and pathogen membranes and direct the appropriate complement response by either promoting the binding of complement activators or inhibitors. The binding of these complement regulators to sugar molecules can vary from location to location, due to their different specificities and because distinct structural and functional subpopulations of sugars are found in different human organs, such as the brain, kidney, and eye. This review will cover recent studies that have provided important new insights into the role of GAGs and sialic acid in complement regulation and how sugar recognition may be compromised in disease.

## Introduction

The complement system plays a vital role in the protection of a host from invading bacteria and other microorganisms. However, this potent immunological weapon must be tightly regulated, or there is a risk of attack of host tissues leading to damage via an inappropriate inflammatory response (1). Sugar molecules provide a diverse and complex means by which the complement system can not only identify bacteria and other invading pathogens as a threat but also identify host surfaces that require protection (2). With three activating pathways of complement, it is the alternative and lectin pathways that utilize sugar molecules the most (1). The lectin pathway is activated by the recognition of carbohydrate moieties, such as mannose or glucose, on the surface of bacteria, by the mannose-binding lectin or ficolins (3, 4). On the other hand, the alternative pathway of complement is modulated in host tissues by glycans such as sialic acid [the predominant form being *N*-acetylneuraminic acid (Neu5Ac)] or the glycosaminoglycan (GAG) chains of proteoglycans. The presentation of specific sialic acid or GAG structures on the surface of a cell, or within the extracellular matrix, can dictate whether positive or negative regulation of an immune response occurs, including complement activation. This is because the sugar compositions of both GAGs and sialic acid can vary greatly from one organ to another and even between different regions/microenvironments within the same tissue (2, 5, 6).

Glycosaminoglycans and sialic acid play an important role in the recruitment and control of a wide range of innate/cellular immune system regulatory proteins, as well as proteins involved in tissue remodeling following an inflammatory response (7, 8). For example, GAGs are key regulators of pulmonary inflammation during lung infection through their binding of cytokines, chemokines, and growth factors, which leads to leukocyte adhesion and accumulation (9). Interestingly, the protein tumor necrosis factor-stimulated gene-6, which plays a role in protecting tissues from the damaging effects of inflammation, has recently been found to antagonize the interaction of the chemokine CXCL8 with the GAG heparan sulfate (HS) on the surface of endothelial cells and thereby inhibit neutrophil extravasation (10). In this mini-review, we will concentrate on the role of sulfated GAGs (particularly HS) and sialic acid on the recruitment and regulation of components of the complement cascade.

## Modulation of Complement by Sulfated GAGs

**Figure 1:**
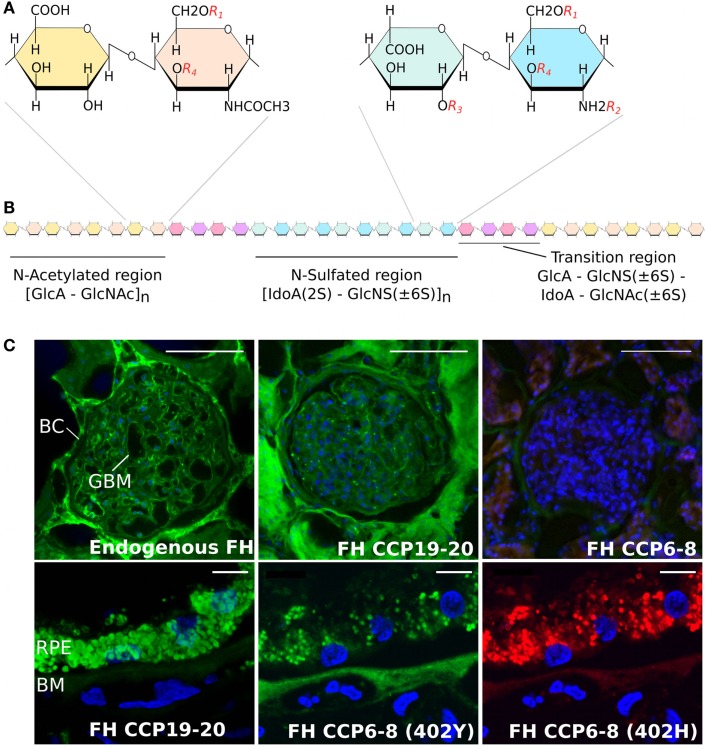
**Structure of heparan sulfate and the binding of factor H to human kidney and eye tissue**. **(A)** Schematic showing disaccharide structures found in the HS chain. These are comprised of glucuronic acid (GlcA) and N-acetylated glucosamine (GlcNAc), found predominately in the N-acetylated region, and iduronic acid (IodA) and N-sulfated glucosamine (GlcNS) that are found in the *N*-sulfated region. The four possible sulfation positions are listed as: *R_1_*, 6-O-sulfation; *R_2_*, N-sulfation; *R_3_*, 2-O-sulfation; and *R_4_*, 3-O-sulfation. **(B)** Diagram demonstrating the distribution of the *N*-acetylated and *N*-sulfated regions of HS and their separation by short transition regions. **(C)** Staining of human kidney glomeruli (top panels) and the macula region of the human eye (lower panels) for endogenous FH and FH CCP6–8 and CCP19–20 binding sites; for full details, see Ref. (21). Endogenous FH (green staining) can be seen in both the Bowman’s capsule (BC) and glomeruli basement membrane (GBM) in the human kidney, where this binding is predominately mediated by the CCP19–20 region of the protein. However, the CCP19−20 region of FH binds poorly to the Bruch’s membrane (BM) of eye, where the interaction of FH is predominantly mediated by CCP6−8. The Y402H polymorphism, found in CCP7, alters the binding of FH to BM, demonstrated by the lack of red staining in the bottom right hand side panel. Scale bars in the top panels of **(C)** represent 100 μm, and in the lower panels represent 10 μm.

There are four different types of sulfated GAGs that are found ubiquitously in human tissues – namely chondroitin sulfate, dermatan sulfate (DS), HS, and keratan sulfate – all of which are attached to proteoglycan core proteins and have considerable diversity in their “sequence” of sugars (11). Of these, HS is the most structurally diverse and plays a vital role in cell differentiation, signaling, and immune homeostasis (12–16). The HS chain comprises repeating disaccharide units of a glucuronic acid (GlcA) or iduronic acid (IdoA) linked to *N*-glucosamine (GlcN) (17, 18). As shown in Figure 1A, each disaccharide has four positions that can be variably modified with sulfation (or acetylation in the case of the *N* position of GlcN) and, along with the epimerization of some GlcA sugars to IdoA, this allows for immense structural diversity of HS chains that are typically 50–200 disaccharides in length. This diversity is made more complex by the subdivision of HS chains into *N*-sulfated (NS) regions and *N*-acetylated (NA) regions (of variable length) separated by small “transition” (NS/NA) zones (see Figure 1B). Overall, it is this complexity that provides a broad range of structures that can be recognized differentially by proteins, such that the biosynthesis of distinct “sequences” at particular tissue sites can promote/regulate their binding within a particular microenvironment (2, 19). For example, the complement regulatory proteins factor H (FH) and factor H-like protein 1 (FHL-1), a truncated version of FH generated through alternative splicing [that has 7 rather than 20 complement control protein (CCP) repeats], prevent inappropriate alternative pathway activation/amplification in host tissues; in part, this is mediated by their binding to HS (and DS) on cell surfaces and within the surrounding matrix (20–22). One particular variant of FH/FHL-1 (termed 402H; that has a histidine at residue 402 in CCP7) is associated with an increased risk of age-related macular degeneration (AMD), a common cause of blindness in developed nations, and requires a high level of HS sulfation for its binding (23, 24). Because such highly sulfated sequences are rare within the human Bruch’s membrane (BM) (an extracellular matrix of the eye), this might be the underlying cause of why complement dysregulation occurs at this site; i.e., due to insufficient FH/FHL-1 binding in 402H individuals (20, 22), leading to local inflammation that drives AMD pathology. FHL-1 has been found to be the major form of FH within BM (22) and unlike FH does not have a second GAG-binding domain (in CCP19–20) to compensate for its impaired tissue recognition; FHL-1 also lacks the sialic acid-binding site in CCP20 (see below). Importantly, the recent finding that the overall amount of HS in BM falls during normal aging (accompanied by a significant reduction in the level of sulfation) might explain the age-related nature of AMD (25); i.e., further impairing binding of the 402H variant of FH/FHL-1. Age-dependent changes in the sulfation patterns of HS have also been reported in tissues such as in the aorta (26) and in outgrowth endothelial cells (27); in the latter, a decrease in the amount of 6*-*O-sulfation with age results in a decrease in the migratory capacity of these cells toward vascular endothelial growth factor and stromal cell-derived factor 1α.

Properdin has an opposing role to FH/FHL-1 in that it is a positive regulator of the complement system (28). Properdin stabilizes the alternative pathway C3 convertase (C3bBb) allowing more conversion of C3 into C3b and thus amplification of complement activation. Because properdin exists as oligomers (dimers, trimmers, and tetramers), which can bind multiple C3b molecules, it can therefore act as a platform for the assembly of additional C3 convertases (29, 30). It has also been demonstrated that properdin can bind to HS and chondroitin sulfate on apoptotic T cells, thereby aiding their clearance by promoting complement-mediated opsonization/phagocytosis (31, 32). Furthermore, it has been shown that properdin and FH bind distinct HS sugars on renal tubular epithelial cells (33, 34) demonstrating the power of GAGs to mediate immune homeostasis on tissues by recruiting both positive and negative regulators of complement through the presentation of different sulfation patterns [reviewed in Ref. (28)].

## Modulation of Complement by Sialic Acid

**Figure 2:**
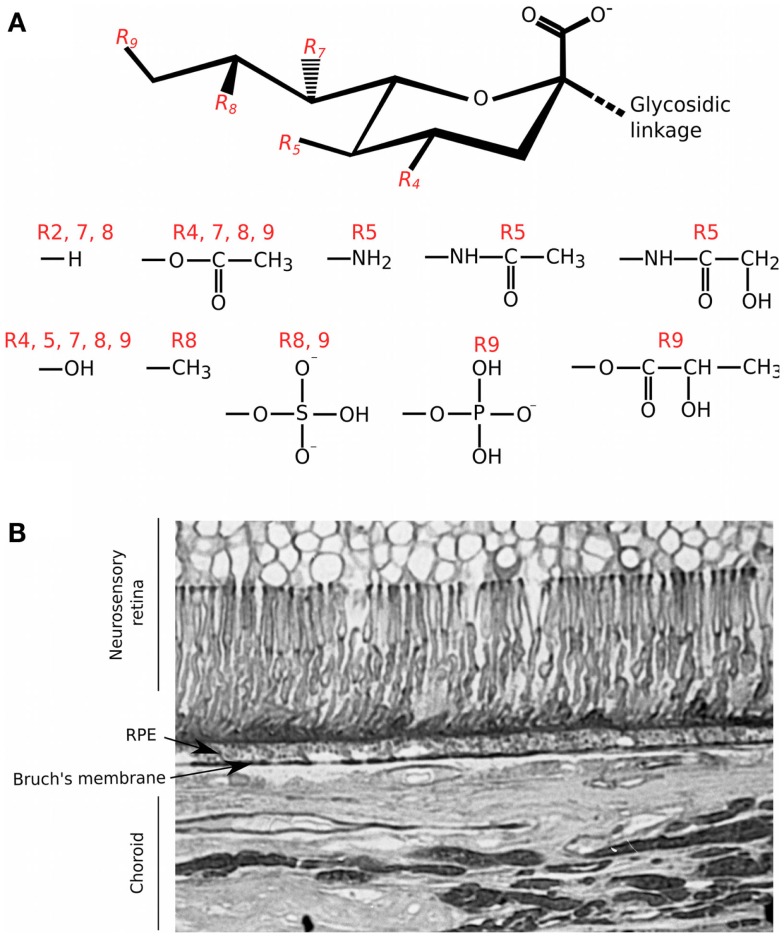
**Structural diversity of sialic acids and their distribution in the human eye**. **(A)** Schematic of the basic 9 carbon structure of sialic acid and some of the possible substitutions (35); it is the C2 position that forms the glycosidic linkage to other saccharides within *O*- and *N*-linked glycan chains. R4, R5, R7, R8, and R9 groups are variably modified with the chemical groups illustrated. **(B)** Staining of a tissue section of human macula with the *Maackia amurensis* (MAA) lectin was carried out as described in Bishop et al. (5); MAA has high affinity for sialic acid linked α2–3 to galactose (Neu5Acα2–3Gal). Staining with MAA is seen throughout different structures of the eye but with particular intensity on Bruch’s membrane.

Sialic acid also mediates complement interactions and this family of sugars is typically found at the termini of the *N*- and *O*-linked glycans substituting mammalian cell surface and secreted proteins (35, 36). The basic nine-carbon structure can be modified at the 4, 5, 7, 8, and 9 positions to generate a large amount of structural diversity (see Figure 2). It is the C2 carbon that forms the glycosidic bond to the neighboring sugar, i.e., at multiple different positions, allowing for variation in its orientation of presentation (35, 37, 38). Like GAGs, sialic acids can also control the activation of complement through binding FH; e.g., on erythrocytes, conferring protection from the spontaneous tick over of the alternative pathway (39). The binding of FH to sialic acid results in an increased affinity for C3b and thus enhances its cofactor and decay accelerating activities. However, FH binding can be influenced by the type and modifications of sialic acid, e.g., 9-O-acetylation of sialic acid reduces the affinity for FH (39, 40). In this regard, the molecular mechanism by which FH can attach to surfaces via sialic acid, while simultaneously binding C3b, has recently been elucidated (41); crystal structure analyses identified the amino acid residues in the CCP20 domain of FH that bind the glycerol side chain (C7–C9) and carboxyl group of *N*-acetylneuraminic acid (Neu5Ac). Furthermore, it was shown that there is a high level of specificity in the interaction of FH with Neu5Ac since this is dependent on the type of glycosidic bond present; i.e., FH binds α2–3, but not α2–6 or α2–8 sialic acid linkages. Mutations in the residues in FH that are responsible for recognizing sialic acid are associated with the rare kidney disease atypical hemolytic uremic syndrome (aHUS) (42). These changes perturb the Neu5Ac binding pocket and reduce the affinity of FH for sialic acid, providing a biochemical explanation for poor complement regulation on the glomerular endothelium in aHUS (41).

The CCP19–20 region of FH is also known to bind HS (43), most likely at an interaction surface overlapping that for sialic acid (41); thus there is the possibility that these sugars might compete for binding, e.g., on cell/matrix surfaces where both are present. Although this sialic acid/GAG-binding site in CCP19–20 does not contribute greatly to FH’s binding to BM (21), it is known that sialic acid is present throughout the human eye (5); this includes Neu5Acα2–3 (see Figure 2B). Therefore, it is possible that sialic acid may contribute to the binding of FH through other, as of yet uncharacterized, sites. Indeed, treatment of eye tissue with HS/DS-degrading enzymes only reduced endogenous FH levels by ~50%, consistent with the possibility that sialic acid could also be mediating binding to structures including BM (20).

## Tissue Specificity

As described above, both GAGs and sialic acid display considerable molecular diversity. However, importantly, there are differences in the populations of structures/sequences of these sugars found within different tissues. For example, HS is thought to play a regulatory role in many physiological processes (13–15) through the tissue-specific (or least tissue-restricted) biosynthesis of particular sulfation patterns as a form of “zip code” [reviewed in Ref. (2, 19)]. Its variations in sequence pattern can even be seen between different regions of the same tissue, as illustrated by the distinct HS epitopes mapped within the human macula (6), within pancreatic islets (44), and in the human kidney (45).

There is also evidence that functional HS “area codes” are different in the human kidney to those found in the human eye (21); i.e., those that mediate FH binding. Like BM, the glomerular basement membrane is an extracellular matrix that protects itself from complement attack by recruiting FH, in this case, through its CCP19–20 domain binding (at least in part) to HS (see Figure 1C). It has been shown previously that, while the CCP19–20 region mediates the binding of FH to glomeruli, it is the CCP6–8 region that is mainly responsible for binding to HS (and DS) in BM (20, 21). This demonstrates a level of specificity in the biosynthesis of functional HS sequences in the different tissues, or alternatively, that the binding specificity of these two regions of FH has become tuned to the different “compositions” of HS found in the two locations. This is also consistent with the observation that mutations in the CCP19–20 region of FH (46, 47), which are mainly associated with aHUS, do not present with an ocular phenotype but frequently effect heparin/HS binding [see Ref. (21, 48–50) for further discussion]. Similarly, the Y402H polymorphism in CCP7 of FH/FHL-1, a major risk factor for AMD, does not predispose individuals to kidney disease.

Transgenic mouse studies demonstrate that knocking out expression of FH causes aHUS (51) as well as some features that resemble AMD (52). Furthermore, by expressing a form of murine FH without CCPs16–20, it was demonstrated that this region of FH is important in the development of aHUS (53); this is consistent with the recent findings that the CCP19–20 region of FH likely plays a critical role in self recognition in kidney glomeruli through its binding of HS and/or sialic acid (21, 41). In fact, it has been proposed that FH is held in an inactive “latent” conformation by intramolecular interactions and upon binding to HS or sialic acid the conformation changes to one that has higher affinity for C3b and increased co-factor activity (54, 55). Therefore, the presence of HS or sialic acid on host cells may regulate not only the localization of FH but also the affinity for C3b.

Infection with enterohemorrhagic *E. coli* can also cause typical (or infection-induced) HUS; the shiga toxin produced by the bacteria can bind directly to the CCP6–8 or CCP18–20 regions of FH and impairs cofactor activity on cell surfaces but not in the fluid phase (56). Thus, it seems likely that surface recognition mediated by these regions of FH is inhibited through their binding to shiga toxin, although this requires further investigation. Similarly, the condition dense deposit disease can be caused by systemic loss of FH, normally due to mutations affecting the protein structure or its secretion. The resulting global dysregulation of complement results initially in progressive nephropathy with dense drusen-like deposits in the glomerular basement membrane, and later with drusen formation in BM of the eye (55, 57). However, the Y402H polymorphism in the HS-binding site of FH is associated with increased risk of dense deposit disease (58), so a role for GAGs (or sialic acid) is not an impossibility, but this coding change does also affect other functional activities of FH [see Ref. (12)].

The exciting work from Blaum and co-workers (41) has demonstrated that the CCP20 region of FH mediates considerable specificity for particular sialic acid structures (i.e., for Neu5Acα2–3), where amino acid residues involved in their recognition are associated with complement dysregulation in the kidney (46, 47). This suggests that there may be parallels with FH’s tissue specificity for GAG binding (21). In this regard, we know that distinct sialic acid structures are present in different parts of the eye, including within BM (5) and, therefore, it will be interesting to see whether different regions of FH differentially recognize sialic acids in a tissue-specific manner.

The brain is another organ where interactions of complement with host sugars have been found to contribute to immune homeostasis and become dysregulated in disease; in this context, it is believed that complement proteins, including FH, are synthesized locally within brain tissue (59). For example, FH has been shown to associate with the brain lesions of Alzheimer’s disease patients through the binding of HS (60), changes in HS structure are associated with disease progression (61). HS has been shown to bind amyloid-β (62) where this is modulated by the level of HS sulfation (63). In fact, it is believed that neurotoxic amyloid-β competes with neuroprotective fibroblast growth factor 2 for a common HS binding site (63). Furthermore, it has been suggested that the presence of amyloid-β prevents the heparanase-mediated turnover of HS chains (64), which could lead to enhanced binding of FH to HS structures within brain lesions, hindering their clearance by complement. HS has also been shown to regulate the processing of the amyloid precursor protein to amyloid-β by the Alzheimer’s beta-secretase, BACE-1 (65). This is mediated via direct binding of HS to this enzyme, where the specificity of the interaction, e.g., with regard to sulfation pattern, has allowed the generation of heparin derivatives and HS oligosaccharides with therapeutic potential for Alzheimer’s disease (66, 67).

The presence of sialic acid on neuronal cells can prevent the activation of the classical complement pathway by masking the binding sites for C1q (68). The removal of sialic acid results in C1q binding, activation of the classical pathway, and opsonization of the neuronal cells with C3b; microglial cells in the brain can then recognize C3b via Complement Receptor 3 (CR3) and activate the phagocytosis of these labeled cells. It has been postulated that the presence of sialic acid on the cell surface acts as a marker of cellular health that may be lost/impaired during inflammation and oxidative stress (69).

## Modulation of the Complement Response by Pathogens

As described already, FH has two HS-binding regions and at least one site for interaction with sialic acid and with its flexible, modular, structure FH is capable of interacting with several self-ligands on the host surface simultaneously (70), which is believed to enhance its binding avidity. This allows for the recognition of a diverse range of cell and tissue types as well as making it harder for microorganisms to recruit FH to avoid host defense. However, the interplay between host and pathogen is like a constant weapons race. It is therefore not surprising that pathogens have evolved ways to mimic these self-associated molecular patterns (SAMPs) (71). Many human pathogens, including *Pseudomonas aeruginosa* have in common with human cells the sialic acid, Neu5Ac, on their surface (72), which allows them to recruit FH from the blood and thereby prevent a complement-mediated response (73). *Neisseria gonorrhoeae* also have surface sialic acid and this was shown to bind FH in the CCP16–20 region (74), and in light of recent discoveries, the sialic acid is likely to bind CCP20 (41). Bacteria either synthesize the sialic acid *de novo* or acquire it from their host by secreting a sialidase enzyme that cleaves sialic acid from host cells, which can then be taken up and presented via bacterial transporters (75). Currently, no pathogens have developed the ability to create sulfated GAGs (71, 76). However, bacteria have developed proteins that mimic host carbohydrates such as *Neisseria meningitides*, which produces a FH-binding protein that has been shown to bind to the CCP6–8 region of FH (77).

## Modulation of the Complement Response by Cancer

Like pathogens, cancer cells can also protect themselves from complement-mediated immune activation (78). FH and FHL-1 expression is up-regulated in some cancers (79) and inhibition of their expression reduces the growth rate of the cells *in vivo* (80). Cancer cells also commonly up-regulate sialic acid synthesis (81), possibly by up-regulating sialyltransferases (78), to reach a state that has been coined “super-self” (82). It is thought that increased surface levels of sialic acid confer protection against complement by recruiting FH (83) – removing sialic acid from cancer cells enhances their complement-mediated lysis (84) – and contributes to immune evasion from NK and other immune cells by non-complement-mediated mechanisms (78). Interestingly, many breast cancer cells have an increased amount of HS proteoglycans on their surfaces compared to normal mammary cells (85), and therefore, it is tempting to hypothesize that the up-regulation of this SAMP, like sialic acid, confers increased protection of cancer cells to complement by recruiting FH.

## Conclusion

The structural diversity of GAGs and sialic acids makes a significant contribution to the regulation of immune homeostasis through the formation of “sugar postcodes” in human tissues. In particular, these sugars represent molecular signals capable of specifically recruiting either complement inhibitors, or activators, to a host surface in a tissue-specific fashion. Recent evidence suggests that changes to the GAG/sialic acid “repertoire” in a particular tissue, whether caused by disease or normal aging, can result in an inappropriate complement response and tissue damage. In some circumstances, it may be possible to correct this dysregulation of the innate immune system; e.g., the use of modified GAGs that interfere with the binding of properdin (but not FH) to HS on renal tubular epithelial cells might be of benefit in proteinuric renal disease (33, 34). As such, drugs aimed at modifying complement–sugar interactions in a tissue-specific manner could represent a viable therapeutic option in a number of disease contexts.

## Conflict of Interest Statement

The authors declare that the research was conducted in the absence of any commercial or financial relationships that could be construed as a potential conflict of interest.

## Abbreviations

AMD, age-related macular degeneration; aHUS, atypical hemolytic uremic syndrome; BM, Bruch’s membrane; CCP, complement control protein; DS, dermatan sulfate; FH, factor H; FHL-1, factor H-like protein 1; HS, heparan sulfate; GAG, glycosaminoglycan; GlcA, glucuronic acid; GlcN, *N*-glucosamine; IdoA, iduronic acid; Neu5Ac, *N*-acetylneuraminic acid.

## References

[1] Nonaka M Evolution of the complement system. Subcell Biochem (2014) 80:31–43 10.1007/978-94-017-8881-6_3 24798006

[2] Langford-Smith A Keenan TDL Clark SJ Bishop PN Day AJ. The role of complement in age-related macular degeneration: heparan sulphate, a ZIP code for complement factor H? J Innate Immun (2014) 6:407–16. 10.1159/000356513 24335201 PMC4086042

[3] Fujita T. Evolution of the lectin-complement pathway and its role in innate immunity. Nat Rev Immunol (2002) 2:346–53. 10.1038/nri800 12033740

[4] Cestari I dos S Krarup A Sim RB Inal JM Ramirez MI. Role of early lectin pathway activation in the complement-mediated killing of *Trypanosoma cruzi*. Mol Immunol (2009) 47:426–37. 10.1016/j.molimm.2009.08.030 19783051

[5] Bishop PN Boulton M McLeod D Stoddart RW. Glycan localization within the human interphotoreceptor matrix and photoreceptor inner and outer segments. Glycobiology (1993) 3:403–12. 10.1093/glycob/3.4.403 8400552

[6] Clark SJ Keenan TDL Fielder HL Collinson LJ Holley RJ Merry CLR Mapping the differential distribution of glycosaminoglycans in the adult human retina, choroid, and sclera. Invest Ophthalmol Vis Sci (2011) 52:6511–21. 10.1167/iovs.11-7909 21746802 PMC3175996

[7] Parish CR. The role of heparan sulphate in inflammation. Nat Rev Immunol (2006) 6:633–43. 10.1038/nri1918 16917509

[8] Rabinovich GA van Kooyk Y Cobb BA Glycobiology of immune responses. Ann N Y Acad Sci (2012) 1253:1–15 10.1111/j.1749-6632.2012.06492.x 22524422 PMC3884643

[9] Gill S Wight TN Frevert CW. Proteoglycans: key regulators of pulmonary inflammation and the innate immune response to lung infection. Anat Rec (Hoboken) (2010) 293:968–81. 10.1002/ar.21094 20503391 PMC4121077

[10] Dyer DP Thomson JM Hermant A Jowitt TA Handel TM Proudfoot AEI TSG-6 inhibits neutrophil migration via direct interaction with the chemokine CXCL8. J Immunol (2014) 192:2177–85. 10.4049/jimmunol.1300194 24501198 PMC3988464

[11] Taylor KR Gallo RL. Glycosaminoglycans and their proteoglycans: host-associated molecular patterns for initiation and modulation of inflammation. FASEB J (2006) 20:9–22. 10.1096/fj.05-4682rev 16394262

[12] Clark SJ Bishop PN Day AJ. Complement factor H and age-related macular degeneration: the role of glycosaminoglycan recognition in disease pathology. Biochem Soc Trans (2010) 38:1342–8. 10.1042/BST0381342 20863311

[13] Bishop JR Schuksz M Esko JD. Heparan sulphate proteoglycans fine-tune mammalian physiology. Nature (2007) 446:1030–7. 10.1038/nature05817 17460664

[14] Turnbull JE. Heparan sulfate glycomics: towards systems biology strategies. Biochem Soc Trans (2010) 38:1356–60. 10.1042/BST0381356 20863313

[15] Langford-Smith A Wilkinson FL Langford-Smith KJ Holley RJ Sergijenko A Howe SJ Hematopoietic stem cell and gene therapy corrects primary neuropathology and behavior in mucopolysaccharidosis IIIA mice. Mol Ther (2012) 20:1610–21. 10.1038/mt.2012.82 22547151 PMC3421066

[16] Meade KA White KJ Pickford CE Holley RJ Marson A Tillotson D Immobilization of heparan sulfate on electrospun meshes to support embryonic stem cell culture and differentiation. J Biol Chem (2013) 288:5530–8. 10.1074/jbc.M112.423012 23235146 PMC3581394

[17] Mulloy B Forster MJ. Conformation and dynamics of heparin and heparan sulfate. Glycobiology (2000) 10:1147–56. 10.1093/glycob/10.11.1147 11087707

[18] Esko JD Selleck SB. Order out of chaos: assembly of ligand binding sites in heparan sulfate. Annu Rev Biochem (2002) 71:435–71. 10.1146/annurev.biochem.71.110601.135458 12045103

[19] Clark SJ Bishop PN Day AJ The proteoglycan glycomatrix: a sugar microenvironment essential for complement regulation. Front Immunol (2013) 4:412 10.3389/fimmu.2013.00412 24324472 PMC3840399

[20] Clark SJ Perveen R Hakobyan S Morgan BP Sim RB Bishop PN Impaired binding of the age-related macular degeneration-associated complement factor H 402H allotype to Bruch’s membrane in human retina. J Biol Chem (2010) 285:30192–202. 10.1074/jbc.M110.103986 20660596 PMC2943316

[21] Clark SJ Ridge LA Herbert AP Hakobyan S Mulloy B Lennon R Tissue-specific host recognition by complement factor H is mediated by differential activities of its glycosaminoglycan-binding regions. J Immunol (2013) 190:2049–57. 10.4049/jimmunol.1201751 23365078 PMC3672945

[22] Clark SJ Schmidt CQ White AM Hakobyan S Morgan BP Bishop PN. Identification of factor H-like protein 1 as the predominant complement regulator in Bruch’s membrane: implications for age-related macular degeneration. J Immunol (2014) 193:4962–70. 10.4049/jimmunol.1401613 25305316 PMC4225158

[23] Clark SJ Higman VA Mulloy B Perkins SJ Lea SM Sim RB His-384 allotypic variant of factor H associated with age-related macular degeneration has different heparin binding properties from the non-disease-associated form. J Biol Chem (2006) 281:24713–20. 10.1074/jbc.M605083200 16787919

[24] Prosser BE Johnson S Roversi P Herbert AP Blaum BS Tyrrell J Structural basis for complement factor H linked age-related macular degeneration. J Exp Med (2007) 204:2277–83. 10.1084/jem.20071069 17893204 PMC2118454

[25] Keenan TDL Pickford CE Holley RJ Clark SJ Lin W Dowsey AW Age-dependent changes in heparan sulfate in human Bruch’s membrane: implications for age-related macular degeneration. Invest Ophthalmol Vis Sci (2014) 55:5370–9. 10.1167/iovs.14-14126 25074778

[26] Feyzi E Saldeen T Larsson E Lindahl U Salmivirta M. Age-dependent modulation of heparan sulfate structure and function. J Biol Chem (1998) 273:13395–8. 10.1074/jbc.273.22.13395 9593669

[27] Williamson KA Hamilton A Reynolds JA Sipos P Crocker I Stringer SE Age-related impairment of endothelial progenitor cell migration correlates with structural alterations of heparan sulfate proteoglycans. Aging Cell (2013) 12:139–47. 10.1111/acel.12031 23190312

[28] Kouser L Abdul-Aziz M Nayak A Stover CM Sim RB Kishore U. Properdin and factor H: opposing players on the alternative complement pathway “see-saw”. Front Immunol (2013) 4:93. 10.3389/fimmu.2013.00093 23630525 PMC3632793

[29] Alcorlo M Tortajada A Rodríguez de Córdoba S Llorca O. Structural basis for the stabilization of the complement alternative pathway C3 convertase by properdin. Proc Natl Acad Sci U S A (2013) 110:13504–9. 10.1073/pnas.1309618110 23901101 PMC3746899

[30] Lesher AM Nilsson B Song WC Properdin in complement activation and tissue injury. Mol Immunol (2013) 56:191–8 10.1016/j.molimm.2013.06.002 23816404 PMC3730815

[31] Kemper C Atkinson JP Hourcade DE. Properdin: emerging roles of a pattern-recognition molecule. Annu Rev Immunol (2010) 28:131–55. 10.1146/annurev-immunol-030409-101250 19947883

[32] Kemper C Mitchell LM Zhang L Hourcade DE. The complement protein properdin binds apoptotic T cells and promotes complement activation and phagocytosis. Proc Natl Acad Sci U S A (2008) 105:9023–8. 10.1073/pnas.0801015105 18579773 PMC2449358

[33] Zaferani A Vivès RR Van Der Pol P Navis GJ Daha MR Van Kooten C Factor H and properdin recognize different epitopes on renal tubular epithelial heparan sulfate. J Biol Chem (2012) 287:31471–81. 10.1074/jbc.M112.380386 22815489 PMC3438980

[34] Zaferani A Vivès RR Van Der Pol P Hakvoort JJ Navis GJ Van Goor H Identification of tubular heparan sulfate as a docking platform for the alternative complement component properdin in proteinuric renal disease. J Biol Chem (2011) 286:5359–67. 10.1074/jbc.M110.167825 21135110 PMC3037648

[35] Varki NM Varki A. Diversity in cell surface sialic acid presentations: implications for biology and disease. Lab Invest (2007) 87:851–7. 10.1038/labinvest.3700656 17632542 PMC7100186

[36] Varki A Sialic acids in human health and disease. Trends Mol Med (2008) 14:351–60 10.1016/j.molmed.2008.06.002 18606570 PMC2553044

[37] Gagneux P Cheriyan M Hurtado-Ziola N van der Linden ECMB Anderson D McClure H Human-specific regulation of alpha 2-6-linked sialic acids. J Biol Chem (2003) 278:48245–50. 10.1074/jbc.M309813200 14500706

[38] Stencel-Baerenwald JE Reiss K Reiter DM Stehle T Dermody TS. The sweet spot: defining virus-sialic acid interactions. Nat Rev Microbiol (2014) 12:739–49. 10.1038/nrmicro3346 25263223 PMC4791167

[39] Varki A Gagneux P. Multifarious roles of sialic acids in immunity. Ann N Y Acad Sci (2012) 1253:16–36. 10.1111/j.1749-6632.2012.06517.x 22524423 PMC3357316

[40] Shi W-X Chammas R Varki NM Powell L Varki A. Sialic acid 9-O-acetylation on murine erythroleukemia cells affects complement activation, binding to i-type lectins, and tissue homing. J Biol Chem (1996) 271:31526–32. 10.1074/jbc.271.49.31526 8940168

[41] Blaum BS Hannan JP Herbert AP Kavanagh D Uhrin D Stehle T. Structural basis for sialic acid-mediated self-recognition by complement factor H. Nat Chem Biol (2015) 11:77–82. 10.1038/nchembio.1696 25402769

[42] Ferreira VP Herbert AP Cortes C McKee KA Blaum BS Esswein ST The binding of factor H to a complex of physiological polyanions and C3b on cells is impaired in atypical hemolytic uremic syndrome. J Immunol (2009) 182:7009–18. 10.4049/jimmunol.0804031 19454698 PMC2696619

[43] Perkins SJ Fung KW Khan S. Molecular interactions between complement factor H and its heparin and heparan sulfate ligands. Front Immunol (2014) 5:126. 10.3389/fimmu.2014.00126 24744754 PMC3978290

[44] Theodoraki A Hu Y Poopulasuntharam S Osteonhof A Guimond SE Disterer P Distinct patterns of heparan sulphate in pancreatic islets suggest novel roles in paracrine islet regulation. Mol Cell Endocrinol (2014) 399:296–310. 10.1016/j.mce.2014.09.011 25224485

[45] Lensen JFM Rops ALWMM Wijnhoven TJM Hafmans T Feitz WFJ Oosterwijk E Localization and functional characterization of glycosaminoglycan domains in the normal human kidney as revealed by phage display-derived single chain antibodies. J Am Soc Nephrol (2005) 16:1279–88. 10.1681/ASN.2004050413 15788473

[46] Oppermann M Manuelian T Jozsi M Brandt E Jokiranta TS Heinen S The C-terminus of complement regulator factor H mediates target recognition: evidence for a compact conformation of the native protein. Clin Exp Immunol (2006) 144:342–52. 10.1111/j.1365-2249.2006.03071.x 16634809 PMC1809651

[47] Józsi M Oppermann M Lambris JD Zipfel PF. The C-terminus of complement factor H is essential for host cell protection. Mol Immunol (2007) 44:2697–706. 10.1016/j.molimm.2006.12.001 17208302 PMC2700862

[48] Lehtinen MJ Rops AL Isenman DE van der Vlag J Jokiranta TS. Mutations of factor H impair regulation of surface-bound C3b by three mechanisms in atypical hemolytic uremic syndrome. J Biol Chem (2009) 284:15650–8. 10.1074/jbc.M900814200 19351878 PMC2708861

[49] Kajander T Lehtinen MJ Hyvärinen S Bhattacharjee A Leung E Isenman DE Dual interaction of factor H with C3d and glycosaminoglycans in host-nonhost discrimination by complement. Proc Natl Acad Sci U S A (2011) 108:2897–902. 10.1073/pnas.1017087108 21285368 PMC3041134

[50] Boels MGS Lee DH van den Berg BM Dane MJC van der Vlag J Rabelink TJ. The endothelial glycocalyx as a potential modifier of the hemolytic uremic syndrome. Eur J Intern Med (2013) 24:503–9. 10.1016/j.ejim.2012.12.016 23357408

[51] Pickering MC Cook HT Warren J Bygrave AE Moss J Walport MJ Uncontrolled C3 activation causes membranoproliferative glomerulonephritis in mice deficient in complement factor H. Nat Genet (2002) 31:424–8. 10.1038/ng912 12091909

[52] Coffey PJ Gias C McDermott CJ Lundh P Pickering MC Sethi C Complement factor H deficiency in aged mice causes retinal abnormalities and visual dysfunction. Proc Natl Acad Sci U S A (2007) 104:16651–6. 10.1073/pnas.0705079104 17921253 PMC2034255

[53] Pickering MC de Jorge EG Martinez-Barricarte R Recalde S Garcia-Layana A Rose KL Spontaneous hemolytic uremic syndrome triggered by complement factor H lacking surface recognition domains. J Exp Med (2007) 204:1249–56. 10.1084/jem.20070301 17517971 PMC2118613

[54] Makou E Herbert AP Barlow PN. Functional anatomy of complement factor H. Biochemistry (2013) 52:3949–62. 10.1021/bi4003452 23701234

[55] Loeven MA Rops AL Berden JH Daha MR Rabelink TJ van der Vlag J. The role of heparan sulfate as determining pathogenic factor in complement factor H-associated diseases. Mol Immunol (2015) 63:203–8. 10.1016/j.molimm.2014.08.005 25246018

[56] Orth D Khan AB Naim A Grif K Brockmeyer J Karch H Shiga toxin activates complement and binds factor H: evidence for an active role of complement in hemolytic uremic syndrome. J Immunol (2009) 182:6394–400. 10.4049/jimmunol.0900151 19414792

[57] Mullins RF Russell SR Anderson DH Hageman GS. Drusen associated with aging and age-related macular degeneration contain proteins common to extracellular deposits associated with atherosclerosis, elastosis, amyloidosis, and dense deposit disease. FASEB J (2000) 14:835–46. 10783137

[58] Abrera-Abeleda MA Nishimura C Frees K Jones M Maga T Katz LM Allelic variants of complement genes associated with dense deposit disease. J Am Soc Nephrol (2011) 22:1551–9. 10.1681/ASN.2010080795 21784901 PMC3148710

[59] Veerhuis R Nielsen HM Tenner AJ Complement in the brain. Mol Immunol (2011) 48:1592–603 10.1016/j.molimm.2011.04.003 21546088 PMC3142281

[60] Strohmeyer R Ramirez M Cole GJ Mueller K Rogers J. Association of factor H of the alternative pathway of complement with agrin and complement receptor 3 in the Alzheimer’s disease brain. J Neuroimmunol (2002) 131:135–46. 10.1016/S0165-5728(02)00272-2 12458045

[61] Bruinsma IB Riet L Gevers T Dam GB Kuppevelt TH David G Sulfation of heparan sulfate associated with amyloid-β plaques in patients with Alzheimer’s disease. Acta Neuropathol (2009) 119:211–20. 10.1007/s00401-009-0577-1 19636575

[62] Buée L Ding W Anderson JP Narindrasorasak S Kisilevsky R Boyle NJ Binding of vascular heparan sulfate proteoglycan to Alzheimer’s amyloid precursor protein is mediated in part by the N-terminal region of A4 peptide. Brain Res (1993) 627:199–204. 10.1016/0006-8993(93)90321-D 8298962

[63] Lindahl B Westling C Gimenez-Gallego G Lindahl U Salmivirta M. Common binding sites for β-amyloid fibrils and fibroblast growth factor-2 in heparan sulfate from human cerebral cortex. J Biol Chem (1999) 274:30631–5. 10.1074/jbc.274.43.30631 10521448

[64] Bame KJ Danda J Hassall A Tumova S. A (1-40) prevents heparanase-catalyzed degradation of heparan sulfate glycosaminoglycans and proteoglycans in vitro. A role for heparan sulfate proteoglycan turnover in Alzheimer’s disease. J Biol Chem (1997) 272:17005–11. 10.1074/jbc.272.27.17005 9202014

[65] Scholefield Z Yates EA Wayne G Amour A McDowell W Turnbull JE. Heparan sulfate regulates amyloid precursor protein processing by BACE1, the Alzheimer’s beta-secretase. J Cell Biol (2003) 163:97–107. 10.1083/jcb.200303059 14530380 PMC2173449

[66] Patey SJ Edwards EA Yates EA Turnbull JE. Heparin derivatives as inhibitors of BACE-1, the Alzheimer’s beta-secretase, with reduced activity against factor Xa and other proteases. J Med Chem (2006) 49:6129–32. 10.1021/jm051221o 17004727

[67] Schwörer R Zubkova OV Turnbull JE Tyler PC. Synthesis of a targeted library of heparan sulfate hexa- to dodecasaccharides as inhibitors of β-secretase: potential therapeutics for Alzheimer’s disease. Chemistry (2013) 19:6817–23. 10.1002/chem.201204519 23553710

[68] Linnartz B Kopatz J Tenner AJ Neumann H. Sialic acid on the neuronal glycocalyx prevents complement C1 binding and complement receptor-3-mediated removal by microglia. J Neurosci (2012) 32:946–52. 10.1523/JNEUROSCI.3830-11.2012 22262892 PMC4037907

[69] Linnartz B Neumann H. Microglial activatory (immunoreceptor tyrosine-based activation motif)- and inhibitory (immunoreceptor tyrosine-based inhibition motif)-signaling receptors for recognition of the neuronal glycocalyx. Glia (2013) 61:37–46. 10.1002/glia.22359 22615186

[70] Morgan HP Schmidt CQ Guariento M Blaum BS Gillespie D Herbert AP Structural basis for engagement by complement factor H of C3b on a self surface. Nat Struct Mol Biol (2011) 18:463–70. 10.1038/nsmb.2018 21317894 PMC3512577

[71] Varki A Letter to the glyco-forum: since there are PAMPs and DAMPs, there must be SAMPs? Glycan “self-associated molecular patterns” dampen innate immunity, but pathogens can mimic them. Glycobiology (2011) 21:1121–4 10.1093/glycob/cwr087 21932452 PMC3150115

[72] Vimr ER Kalivoda KA Deszo EL Steenbergen SM Diversity of microbial sialic acid metabolism. Microbiol Mol Biol Rev (2004) 68:132–53 10.1128/MMBR.68.1.132 15007099 PMC362108

[73] Khatua B Ghoshal A Bhattacharya K Mandal C Saha B Crocker PR Sialic acids acquired by *Pseudomonas aeruginosa* are involved in reduced complement deposition and siglec mediated host-cell recognition. FEBS Lett (2010) 584:555–61. 10.1016/j.febslet.2009.11.087 19945458 PMC3640159

[74] Ram S Sharma AK Simpson SD Gulati S McQuillen DP Pangburn MK A novel sialic acid binding site on factor H mediates serum resistance of sialylated *Neisseria gonorrhoeae*. J Exp Med (1998) 187:743–52. 10.1084/jem.187.5.743 9480984 PMC2212180

[75] Severi E Hood DW Thomas GH Sialic acid utilization by bacterial pathogens. Microbiology (2007) 153:2817–22 10.1099/mic.0.2007/009480-0 17768226

[76] DeAngelis PL Microbial glycosaminoglycan glycosyltransferases. Glycobiology (2002) 12:9–16 10.1093/glycob/12.1.9R 11825882

[77] Schneider MC Prosser BE Caesar JJE Kugelberg E Li S Zhang Q Neisseria meningitidis recruits factor H using protein mimicry of host carbohydrates. Nature (2009) 458:890–3. 10.1038/nature07769 19225461 PMC2670278

[78] Büll C den Brok MH Adema GJ. Sweet escape: sialic acids in tumor immune evasion. Biochim Biophys Acta (2014) 1846:238–46. 10.1016/j.bbcan.2014.07.005 25026312

[79] Junnikkala S Jokiranta TS Friese MA Jarva H Zipfel PF Meri S. Exceptional resistance of human H2 glioblastoma cells to complement-mediated killing by expression and utilization of factor H and factor H-like protein 1. J Immunol (2000) 164:6075–81. 10.4049/jimmunol.164.11.6075 10820293

[80] Ajona D Hsu Y-F Corrales L Montuenga LM Pio R. Down-regulation of human complement factor h sensitizes non-small cell lung cancer cells to complement attack and reduces in vivo tumor growth. J Immunol (2007) 178:5991–8. 10.4049/jimmunol.178.9.5991 17442984

[81] Häuselmann I Borsig L. Altered tumor-cell glycosylation promotes metastasis. Front Oncol (2014) 4:28. 10.3389/fonc.2014.00028 24592356 PMC3923139

[82] Macauley MS Paulson JC Immunology: glyco-engineering “super-self”. Nat Chem Biol (2014) 10:7–8 10.1038/nchembio.1415 24346034 PMC3935795

[83] Gancz D Fishelson Z. Cancer resistance to complement-dependent cytotoxicity (CDC): problem-oriented research and development. Mol Immunol (2009) 46:2794–800. 10.1016/j.molimm.2009.05.009 19501402

[84] Donin N Jurianz K Ziporen L Schultz S Kirschfink M Fishelson Z. Complement resistance of human carcinoma cells depends on membrane regulatory proteins, protein kinases and sialic acid. Clin Exp Immunol (2003) 131:254–63. 10.1046/j.1365-2249.2003.02066.x 12562385 PMC1808622

[85] Gomes AM Stelling MP Pavão MSG. Heparan sulfate and heparanase as modulators of breast cancer progression. Biomed Res Int (2013) 2013:852093. 10.1155/2013/852093 23984412 PMC3747466

